# Contribution of the Semiological Approach to Deixis–Anaphora in Sign Language: The Key Role of Eye-Gaze

**DOI:** 10.3389/fpsyg.2020.583763

**Published:** 2020-11-06

**Authors:** Brigitte Garcia, Marie-Anne Sallandre

**Affiliations:** Structures Formelles du Langage Laboratory, UMR 7023, Centre National de la Recherche Scientifique, University of Paris 8 – University Paris Lumières, Paris, France

**Keywords:** sign language, reference, deixis–anaphora, eye-gaze, enunciation, corpus, typology, highly iconic constructions

## Abstract

We address the issue of deixis–anaphora in sign language (SL) discourse, focusing on the role of eye-gaze. According to the Semiological Approach, SL structuring stems from a maximum exploitation of the visuo-gestural modality, which results in two modes of meaning production, depending on the signer’s semiotic intent. Involving both non-manual and manual parameters, the first mode, expressing the intent to *say while showing*, uses constructions based on structures, the termed “transfer structures.” The second one, expressing the intent to *say without showing*, involves lexical, pointing and fingerspelling units. In order to situate our descriptive concepts with respect to those used by SL linguists who, like us, adopt a cognitive–functionalist perspective, we expose a specific theoretical foundation of our approach, the “enunciation theories.” The concept of “enunciation” is decisive for understanding the role of eye-gaze, as being at the foundation of deixis and the key vector of referential creation and tracking in SL discourse. “Enunciation” entails the opposition between “Enunciation” and “Utterance” Domains. The first links, as co-enunciators, the signer/speaker and his/her addressee, establishing them by the very “act of enunciation” as 1st and 2nd person. The second is internal to the discourse produced. Grounding on corpora of narratives in several SLs (some with no historical link), we illustrate this crucial role of eye-gaze and the diversity of functions it fulfills. Our analyses, carried out in this perspective, attest to the multiple structural similarities between SLs, particularly with regard to transfer structures. This result strongly supports the typological hypothesis underlying our approach, namely, that these structures are common to all SLs. We thus show that an enunciative analysis, based on the key role of eye-gaze in these visual languages that are SLs, is able to give the simplest account of their own linguistic economy and, in particular, of deixis–anaphora in these languages.

## Introduction

Following Apothéloz and Pekarek Doehler (2003, 110), reference can be defined as “the relationship that language maintains with its external environment (whether it is called ‘mental representation,’ ‘world,’ or ‘reality’)”, and the action of referring as drawing attention to an entity—of which the deixis is the vector par excellence. Here we address the issue of deixis and anaphora expression in sign language (SL) discourse and the shared attention processes that underlie it (e.g., Lyons, 1977; Cornish, 2014; Sidnell and Enfield, 2016).

What is at stake is precisely to highlight the central and specific role of the interlocutors’ eye-gaze (both the signer’s and his/her addressee’s) at the very basis of the deixis in these face-to-face languages. To explain the link between eye-gaze and deixis–anaphora in SL—a link that is, in our view, specific to SL—we must first describe our theoretical framework, known as the Semiological Approach (e.g., Cuxac, 1985, 1999, 2000; Fusellier-Souza, 2006, 2012; Cuxac and Sallandre, 2007; Cuxac and Antinoro-Pizzuto, 2010; Garcia and Sallandre, 2014). We focus in particular on our original modeling of highly iconic constructions, typically described in SL literature as “non-conventional” (encompassing terms such as *classifiers predicates/constructions*, *productive signs* or *depicting signs* on the one hand, and *role shifts*, *surrogates*, *enactments*, *constructed actions or dialogues* on the other), which contrast with “conventional units” (that is, lexical units, fingerspelling, and mouthing).

After a long domination of formalist approaches^1^, the study of SL linguistics slowly began to diversify theoretically primarily in the 1990s, and more so in the 2000s (e.g., Stokoe, 1991; Engberg-Pedersen, 1993, 2003; Armstrong et al., 1995; Liddell, 1995, 2003; Wilcox and Wilcox, 1995; Johnston and Schembri, 1999, 2007; Pizzuto and Volterra, 2000; Woll and Sutton-Spence, 2005; Pizzuto et al., 2007). A growing number of SL linguists are adopting, as we are, a cognitive–functionalist perspective. In this context, following the work on ASL by Winston (1991, 1995), Metzger (1995), and especially by Liddell (2003), the non-conventional constructions mentioned, which are highly iconic and therefore for a long time kept on the margins of SL modeling, have aroused a strong revival of interest. Nowadays, these constructions are the object of numerous studies, especially with respect to their role in the expression of reference and referential cohesion. Yet, as shown below, highly iconic constructions have been at the heart of the Semiological Approach from its inception, where they were described early on as part of the set known as Transfer Structures (Cuxac, 1985, 1999). They have been shown to play a central role in doing reference and for referential cohesion, in LSF (Cuxac, 1999, 2000; Sallandre, 2003; Jacob, 2007; Garcia and Sallandre, 2014), in LIS (Pizzuto, 2007), and in other SLs, considered from a comparative typological perspective (Pizzuto et al., 2008; Sallandre, 2014; Sallandre et al., 2016).

However, a difficulty is that, beyond a number of proximities, there are significant discrepancies between our respective ways of segmenting and analyzing these non-conventional constructions, i.e., between our “transfer units” and particularly the constructions that are described as “depicting signs” on the one hand, and “constructed actions/dialogues” (CA/CD) on the other. A theoretical dimension of our approach helps to explain these specificities, namely, the fact that we have opted, from the outset, for “enunciation theories,” a conception of language that developed in Europe in the 1960s and 1970s—and more particularly in France (e.g., Jakobson, 1957; Benveniste, 1966, 1970, 1974; Ducrot, 1972, 1980; Lyons, 1977; Culioli, 1990, 1995). We will therefore recall the main lines of this conception.

We begin with an overview presenting some of the central aspects in the study of reference both in spoken language (SpL) and in SL. We then present our theoretical framework, the Semiological Approach. We next underline and illustrate its typological scope through twelve SLs. Finally, we expose the contribution of the European “enunciation theories” which are inherent to our approach and we deem particularly appropriate for understanding the key role played by the eye-gaze in the expression of SL deixis and more generally in the creation of reference and maintenance of referential cohesion in SL.

## Reference, Deixis, and Anaphora: Background

The linguistic literature on reference and on the resources used by languages to introduce, maintain, and reintroduce an entity is obviously considerable, both for SpL and SL, and cannot be fully presented here. For a long time, however, the work has only concerned SpLs, which moreover were seen as monomodal and, initially at least, in their written form.

### Reference in Spoken Language

A significant part of the discussion on reference in SpL has focused on the respective limits of deixis and anaphora and whether the distinction between these two major sources of reference is relevant (for an overview, see for example Apothéloz and Pekarek Doehler, 2003; Lombardi Vallauri, 2007), the deixis having first been thought to refer to an entity in the extralinguistic context (exophoric reference) while the anaphora would refer to an entity already introduced in the text/discourse (endophoric reference), referred to as its antecedent. Many authors (among them already Lyons, 1977) have in fact shown that in many cases it is difficult to identify such an antecedent and even argued that the addressee can reach the intended anaphoric interpretation without having to identify an antecedent (e.g., Cornish, 1990; Apothéloz, 1995; Croft, 2003). Rather, the addressee should rely on the representation he developed of the referent, which is often difficult to classify as based on prior discourse (the text) or on external context (exophoric). After Fillmore (1975), Levinson (1983) proposed a distinction between two deictic uses: *gestural deixis*, whose interpretation necessarily requires the establishment of physical links with the communicative situation, and *symbolic deixis*, whose interpretation requires only “the knowledge of the basic spatio-temporal parameters of the speech event, of the participants’ roles, their social relationships, and some notions about the preceding discourse” (Lombardi Vallauri, 2007, 312). However, it often proves irrelevant to distinguish the (symbolic) deixis from the anaphora on the basis that deixis would refer to the introduction of the referent, insofar as the latter can be salient in the universe of discourse even if it is not physically present in the situation. In other words, attention is drawn to the difficulty of dissociating extralinguistic context and “text”—together contributing to the “universe of discourse”—and, thus, of distinguishing deixis and anaphora. This would explain why languages use the same resources in both processes, as mentioned by Lyons (1977), Cornish (1990), and Apothéloz (1995). This is the conclusion reached by Lombardi Vallauri (2007, 334), which we will adopt here, motivating our choice to use the undifferentiated term *deixis–anaphora*.

Models have thus moved from the traditional textual approach focusing on the notion of antecedent, to cognitive approaches opening to the concepts of degrees of salience, informational, memorial, and inferential mechanisms, with increasing consideration of mental representations constructed by and from discourse. According to this cognitive–informational paradigm, anchored in a functional conception of language, the mental representations of the interlocutors are dynamic, resulting from the ongoing discourse, the context, and the shared knowledge. The focus is therefore on the search for what conditions the choice of one or another referential expression, this choice being rather considered as relative to the cognitive status of the referents (to their activation status). This concept has led to various models, notably Accessibility Theory, developed by Ariel (1988, 1990). According to Ariel, the speaker chooses a referential expression depending on the degree of accessibility (cognitive or memory status) he/she assumes the addressee attributes to the referent. The lower the referent’s accessibility, the higher the informational content provided by the chosen referential expression (and thus its “phonological weight”), and vice versa. Thus, markers of high accessibility include clitic pronouns, unstressed pronouns, and zero forms, while definite NPs and proper names, with descriptive content and higher phonological weight, are analyzed as markers of referents with low accessibility.

These cognitive–informational approaches are highly relevant in the study of reference today, regardless of language type. The main criticism against them involves the nature of the data, namely, monolog (rather than interactional) sequences, tending to narrative and written texts. Various authors (mostly in SpL linguistics) have shown that with the addition of data from ecological corpora of face-to-face interactions, factors such as cognitive accessibility of the referent and their attentional status are no longer sufficient to account for the choice of one referential expression over another (for an overview of this issue, see Apothéloz and Pekarek Doehler, 2003). We will not elaborate on this point, for lack of space, and given that the data that SL linguists are working on (including ourselves) are mainly monologs and narratives as well. However, we raise this issue as a point of consideration, all the more so, given that SLs are quintessentially face-to-face languages.

### Reference in Sign Language

For SLs, which were introduced into the linguistic discipline much more recently, the study of reference followed the epistemological evolution described in the introduction. Initially seeking to find similarities with SpL, SL studies most often described processes of a nominal (lexical) and pronominal nature—the latter corresponding to a series of visual indexes considered as analogous to SpL pronouns—but also spatial modifications of certain predicates, which were likened to verbal person inflection.

Regarding the reference to an entity present in the situation, the manual pointing (closed fist, index finger fully or partially extended) is very early analyzed in the SLs studied as the main means of creating a reference in a personal (1st, 2nd, or 3rd person) or demonstrative pronoun function (e.g., Friedman, 1975; Deuchar, 1984; Johnston, 1991; Engberg-Pedersen, 1993; Sutton-Spence and Woll, 1999; Cuxac, 2000). Another common description concerns transitive predicates characterized by a directional movement between their two points of articulation (*directional*/*agreement verbs*). The distinction of persons can be signaled by these spatial modifications, marking a location close to the signer for the 1st person, close to the addressee for the 2nd person, or close to another referent present for the 3rd person. Concerning the reference to entities and events not present in the situation, several processes have been identified too across various SLs: the lexical sign referring to the entity is associated with a point in the signing space (termed “locus”) either by modification of its own location (direct spatialization of the sign) or through the creation of this locus via manual pointing, often accompanied by a look at the locus. The locus therefore marks the entity and can be reactivated by the same processes (spatialization of the lexical sign, manual pointing, and possibly the eye-gaze).

However, alongside these nominal and pronominal processes and the use of space, SL linguists soon noted that “non-conventional constructions” also played a role for doing reference (e.g., from various perspectives, Kegl, 1976; Wilbur, 1979; Pizzuto, 1986; Collins-Ahlgren, 1990; Brennan, 1992). The first major type of such constructions was described under the initial designation *classifier constructions* or *classifiers predicates* (Supalla, 1978). The abundant literature on these constructions begins with Frishberg (1975) and Kegl and Wilbur (1976), who mention manual handshapes that vary depending on salient properties of the referents. The study of “handshapes classifiers” is extended to the study of the complex manual constructions of which they are part and which are described as differing from conventional (lexical) signs: the handshape is supposed to refer iconically to a particular class of referents, and the movement represents iconically either the displacement and/or location of the entity, or the way in which the entity is handled, or the size or contours of the shape, by tracing. Those manual constructions are subsequently the object of multiple debates under a wide range of terms impossible to reproduce here (for an overview, see Schembri, 2003).

During the 1980s, another type of construction was described in which the signer takes on the role of one of the discourse entities and which is defined as the privileged means of expression of reported discourse. This phenomenon was first termed “role shifting” following Mandel (1977), then more commonly known as “role shift.” Role shift, described as marked mainly by a movement of the shoulders and by facial expression, is primarily analyzed, within formal approaches, as allowing the expression of a change in point of view, implying changes to the frame of reference, and thus a rearrangement of the loci associated with the referents (e.g., Padden, 1986, 1990; Lillo-Martin and Klima, 1990). With the exception of Mandel and DeMatteo (in Friedman, 1977), the consequences of this “shift” on the referential framework constitute all that is said of these constructions in the literature until the early 1990s. However, starting in the 1990s, some authors, working on corpora of narratives and generally hostile to formalist approaches, broadened the scope of thinking by stressing that the role shift also makes it possible to report not only dialogues but referred entity’s actions, states or thoughts (Smith et al., 1988; Ahlgren, 1990; Meier, 1990). Winston (1991, 1995) proposes a new term, *constructed actions* (CA), for these constructions in which the narrator reproduces the actions of one of the protagonists of the utterance (or of himself at an earlier point), because, she says, this is not simply a copy but a selective reproduction of the reported action by the narrator.

### Reference and Multimodality of Human Language

A notable development in the study of reference both in SL and in SpL occurred at the end of the 1990s, in parallel with the development of models of cognitive accessibility and, more broadly, the rise of cognitive grammars and usage-based grammars. This evolution is fundamentally linked to the introduction of studies on gesture to the linguistic discipline, specifically, the work of Kendon (1988) and McNeill (1992). These authors renewed the field by advocating for a broader conception of human language, whereby any language, SpL or SL, should be seen as a multimodal and multi-semiotic integration.

With regard to SL linguistics, Liddell (1995, 2003) has been the figurehead for this multimodality paradigm. His new descriptive concepts had a significant impact on how these “non-conventional constructions” were going to be taken into account in the analysis of reference in SL. The key point for Liddell is that, whatever the language, SpL or SL, human communication, when considered in the face-to-face interaction, is not confined to the “symbolic” but resorts to other semiotics, such as indexicality and iconicity. It is therefore a question of integrating as such the at least partially “gradient,”^2^ i.e., according to Liddell, “non-grammatically specified” character of certain categories of SL signs, which, he argued, have remained problematic in the literature.

The first of these are the pointing units referred to as “pronouns,” and the so-called agreement verbs (which he renames “indicating verbs”). Relying on *conceptual integration theory* (Fauconnier and Turner, 1996), Liddell analyzes both types as *conceptual blends* of linguistic components (i.e., integrated into the ASL lexicon) and a *gestural* component (i.e., “non-grammatical”). More generally, the set of “directionality” phenomena must, according to him, be understood as pure pointing *gestures* directed toward those spatially grounded conceptual entities that are always “referents,” whether they are physically present in the signing space or discourse constructs. He then proposes the same analysis for classifier constructions, which he renames “depicting verbs.” In his view, these constructions are also a mix of two types of components, a lexically specified component (mainly the handshape) and a gestural component (movement and location). Thus, the unifying property of depicting and indicating verbs would be that both are “directional,” i.e., indexical, the cause of this specificity being the possibility for the articulators used in SL to be oriented in the signing space while conveying a symbolic content. The only difference between the two categories would be that “(…) the directionality of depicting verbs depicts topographical locative information while the directionality of indicating verbs identifies entities” (Liddell, 2003, 268). According to Liddell, depicting verbs constitute a long but finite list of manual constructions. While a full inventory remains to be achieved, it would be possible to describe them as a “large semi-productive derivational system” (Liddell, 2003, 274) based on verbal roots. Finally, following Winston (1991) and Metzger (1995), Liddell extends his application of conceptual blend theory to CA. He characterizes these as a specific type of “blend,” noted for the fact that the signer is a part of it, thus creating what he terms a “surrogate blend.” Like indicating verbs and depicting verbs, any part of the CA that does not involve grammatically specified signs, i.e., for him, anything that involves “gradience,” is considered as gestural.

Ferrara and Hodge (2018), following Liddell and building on Enfield (2009, 2013) concept of *composite utterance*, take up Clark (1996) tripartition and propose that any language production (in SpL or SL) can be analyzed according to three “methods of signaling” (*describing*, *indicating*, and *depicting*), which can be used separately or jointly. For Ferrara and Hodge, this distinction intersects with another one that seems increasingly more widely accepted, especially among authors adopting Liddell’s approach (e.g., Johnston and Schembri, 1999, 2007; Johnston, 2008, 2012, 2019; Cormier et al., 2013; Hodge et al., 2019). The distinction incorporates the types of signs in a continuum from lexical to non-lexical: fully lexical (highly conventionalized) signs; partly lexical signs—which include pointing signs and indicating verbs (cf. agreement verbs), both characterized by their indicative dimension, as well as depicting signs (cf. classifiers constructions) which combine indicating and depicting; and finally non-lexical signs, the “enactments” or “constructed actions/dialogues” (cf. role shifts). The latter “do not have properties of conventionalized symbolism, i.e., meanings that are additional or predictable from the value of their form given a particular context” (Hodge et al., 2019, 36). The recent study by Hodge et al. (2019) on reference in Auslan (Australian SL) adopts this theoretical framework. The authors set out from what they see as a consensus in the literature: that in accordance with the predictions of accessibility theory, both signers and speakers would choose the most informative and phonologically heavy expressions (particularly fully lexical noun phrases) to introduce new referents and, conversely, would favor high accessibility markers (pronouns or zero anaphora) for referents with a high degree of conceptual discourse salience. The study aims to determine to what extent other factors influence the choice of referential expressions, as in particular motivated use of space, animacy, and semiotic form.

The authors statistically analyze tokens of referring expressions in a large corpus of narratives in Auslan. Their results confirm the role of activation status in the choice of referring expression. New referents are phonologically heavier (according to the authors’ definition of “phonological weight,” i.e., combining more diverse semiotics); above all, they use relatively more conventional forms (lexical signs, fingerspelling, and mouthing) than with reintroduced or maintained referents, which involve fewer semiotics and less conventional forms (depicting signs and surrogates/enactments/CA). In addition to activation status, animacy has a significant effect on the number and on the nature of the strategies chosen by signers for each referring expression. Human referents require the fewest semiotics overall; however, animate referents (humans and animals) tend to be phonologically heavier than inanimates when reintroduced. Finally, according to the authors, these various results call into question the assertion that the newer a referent (i.e., the less cognitively accessible), the more informative its expression would be. In fact, non-conventional semiotic strategies such as depicting signs, visible and invisible surrogates, those tend to be used more frequently for reintroduction and maintained reference, are particularly rich in information.

Our overview of the literature is far from being exhaustive. However, we have intentionally excluded previous work (mentioned in the introduction) on reference in LSF, LIS (Italian SL), ASL, and other SLs. These studies, carried out within the Semiological Approach framework, had produced results somewhat similar to those achieved by Hodge et al. (2019) for Auslan. In order to account for this more precisely, we must first present our theoretical framework, which we do in the next section.

## The Semiological Approach to Sign Language

Our conception of SL, designated in recent years as the Semiological Approach, was used to describe LSF (e.g., Cuxac, 1985, 1999, 2000; Cuxac and Sallandre, 2007; Sallandre, 2007; Cuxac and Antinoro-Pizzuto, 2010; Garcia and Sallandre, 2014; Garcia et al., 2018), but other SLs as well: see Pizzuto et al., 2008; Sallandre et al., 2016 for crosslinguistic comparison of various institutional SLs, but also Fusellier-Souza, 2004, 2006, 2012; Martinod, 2019; Martinod et al., 2020, for the description of family and micro-community SLs.

This model was progressively developed from the early 1980s on the basis of close, frame-by-frame, analysis of long discourse corpora, recorded *in situ* (Cuxac, 1985, 1993, 1999). The methodological decision to work on corpora^3^, setting out from a functional and therefore semantically centered perspective (a top-down approach), was original at the time (and remained so until the 1990s), as research on other SLs had long been focused primarily on elicited data such as decontextualized sentences. Cuxac’s preliminary description, setting out from meaning and systematically seeking what conveys this meaning, takes into account from the very beginning all articulators, both manual and non-manual, focusing in particular on the role of eye-gaze (soon established as central). Very early on, he hypothesized that the modality has a strong impact on the structural and typological characteristics of SLs and that the close similarities between them are significant in this respect.

**FIGURE 1:**
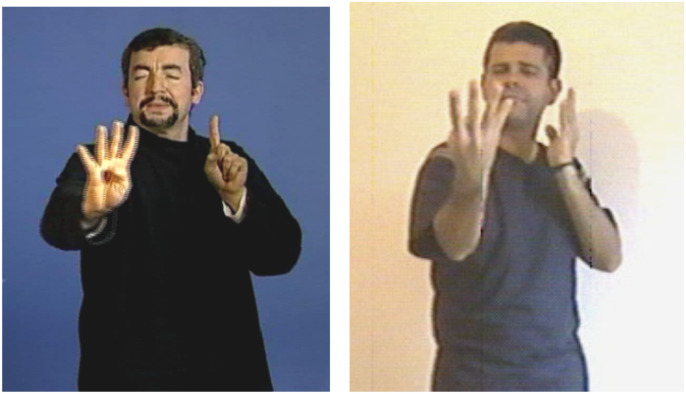
Transfer of size and shape “thin, vertical, elongated shapes” for the referent “fence” in LSF and Libras.

**FIGURE 2:**
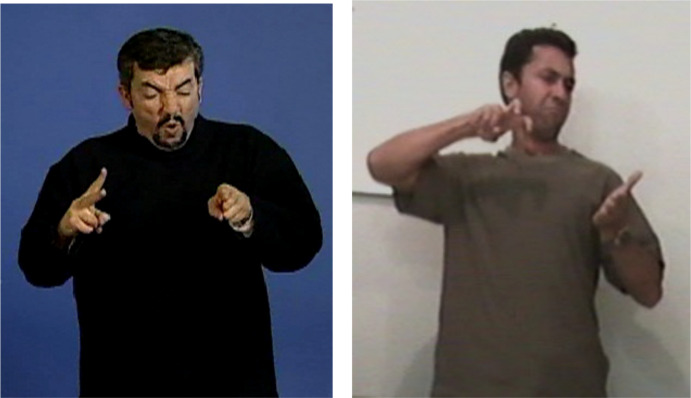
Situational transfer “jump over the fence” in LSF and Libras.

**FIGURE 3:**
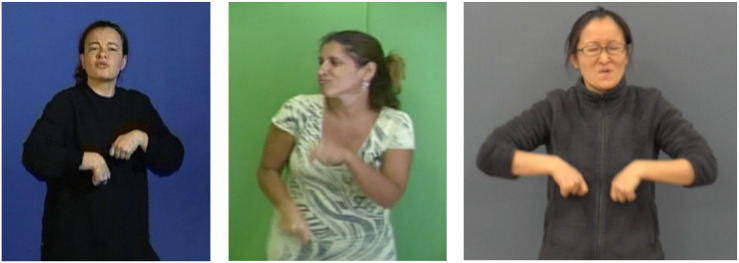
Personal transfer “the horse galloping” in LSF, LIS, and NS.

Like Jouison (1986/1995), the other pioneering LSF linguist, Cuxac soon highlighted the high frequency of highly iconic constructions, which could not be analyzed in the terms of lexical signs. Although involving the same types of manual components as lexical units, these constructions did not meet the criteria then used to define “verbal,” i.e., they were iconic, their meaning varying continuously as their form changed. Focusing on these constructions, Cuxac succeeded in establishing that they stem from a few linguistic structures (or “patterns”), which he calls “transfer structures” (Cuxac, 1985). These structures indeed make it possible to account for the multitude of highly iconic constructions observed in discourse, therefore termed as “transfer units.” The three main transfer structures are the following: the “Size and Shape Transfer” (SST), which allows to show the shape and/or the size of an entity (Figure 1); the “Situational Transfer” (ST), which is to show an actant (dominant hand) moving with respect to a stable locative (typically the non-dominant hand), the scene being represented as a global view, from a distance (external point of view) (Figure 2); and the “Personal Transfer” (PT), by which the signer literally takes on the role of the entity he refers to, and thus shows, as in a close-up shot, the actions it performs or suffers (internal point of view) (Figure 3)^4^. Any transfer unit built from these structures, simultaneously involves all parameters, manual and non-manual. These constructions are *verbal* (that is, linguistic) precisely because they are based on structures, that is, they are composed of constrained elements that fit into paradigms. Another key point which we return to below is that transfer structures share a formal feature, the breaking of eye-gaze toward the addressee.

The characteristic of transfer units is that their global meaning, with a specifying value, comes down to the sum of the (iconic) meaning of their components. However, it must be emphasized that, while it is true that the precise meaning of a transfer unit depends on the context, as frequently noted in the literature, these units have in themselves a highly generic semantic value. Thus, the situational transfer unit in Figure 2 (right image) shows a “shape with double salience moving along an arched path toward a horizontal flat shape.” The transfer structures actually reveal a specific way of saying, their mode of meaning production being, directly, iconicity.^5^

It must be underlined that what is described as “classifier construction”/“depicting sign” in literature only matches the manual component of our transfer units. For Liddell (2003) and the SL linguists who follow his theoretical framework (as Hodge et al., 2019), “depicting verbs/signs” are thus merely manual signs. For us, these manual elements are only one *component* of many in much bigger structures, the transfer structures, which incorporate all parameters, manual and non-manual alike. It should be stressed that a notable difference is the attention we pay to non-manual parameters in the identification of structures and unit types and thus in the choice of linguistic tags. The handshape present within the transfer units is called “proform” in the Semiological Approach. Far from categorizing the referent, proforms constitute a closed list of handshapes that aim to show some aspect of the referent^6^ (Cuxac, 2000).

Transfer units are extremely frequent in discourse, representing up to 70% of occurrences in narratives and up to 30% in other genres, prescriptive and argumentative (*cf.* Sallandre, 2003; Sallandre et al., 2019). They intertwine with the other main type of units, the lexical units, but also with fingerspelling and pointing units. The lexical units have the same mode of meaning production as the SpL words, that is, pure convention. The lexical meaning being mainly carried by the manual components, these units, having a conventional global meaning and a generic value, are instantiated in discourse by a pluri-linear organization of non-manual, semantically specialized parameters: the gaze (rector of interaction and activator of deixis, see section “Enunciation and Deixis-Anaphora: Key Role of Eye-Gaze”), facial expression (carrying aspectual and modal values), facial movements (ensuring a phatic function), and body movements (marking phrases, coordination, and thematic organization). The termed “standard” structures, which involve lexical, pointing, and fingerspelling units, are mainly characterized by the use of signing space in order to create references and to express various semantic relations between referents. Being part of the classical mode of “saying,” the lexical units and the standard structures in which they are employed were essentially the focus of linguistic research on other SLs in the first three decades (1960–1990). Although non-conventional units have been widely studied since the late 1990s, the core of the “grammatical” system is still considered to be the lexical units and the structures that employ them (e.g., Goldin-Meadow and Brentari, 2017; Fenlon et al., 2018).

From our point of view, the space used in the transfer units is itself iconic. It is an imagistic iconicity, showing the deployment of a shape in size and shape transfer, the moving of the actant in situational transfer, the space of action of the transferred entity in personal transfer. However, the space in which the lexical units are used is also iconic. Yet, it is a different type of iconicity, a diagrammatic one (in the Peircean sense)^7^. Transfer units most often have the same format as lexical units (they coincide overwhelmingly with a “minimum unit of realization”) and involve the same types of parametric components^8^. They can also combine with each other, with a lexical unit, or with a manual pointing, depending on regular patterns that result in greater structural complexity. We precise and illustrate these points and the most frequent of these combinations in the next section.

**FIGURE 4:**
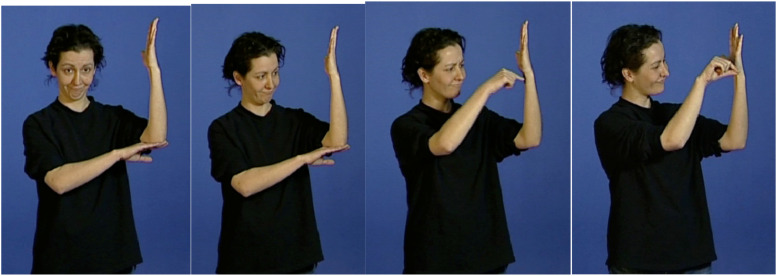
Referential frame switching through the gaze; the tree sequence (corpus LS-COLIN, Cuxac et al., 2002).

However, a central aspect of the Semiological Approach still needs to be clarified, since it gives the model its explanatory dimension. We indeed hypothesize that transfer structures would be found across all SLs around the world. This hypothesis was first supported by the analysis of *homesigns*, family sign languages developed by deaf children isolated in hearing environments (Goldin-Meadow, 1991, 2003), and the description of SLs developed in ontogenesis by deaf adults in Brazil, without contact with any institutional SL (see Fusellier-Souza, 2004, 2006, 2012, and more recently, Martinod, 2019; Martinod et al., 2020)^9^. In these SLs, created at the initiative of the deaf themselves and developed over their lifetime, these studies have found the same transfer structures. They coexist alongside standard structures, just like in institutional macro-community SLs. These observations form the basis for our proposed scenario for SL semiogenesis. Starting from an initial mode of saying based (as *in spite of* signers themselves) on an iconicization of their perceptivo-practical experience, transfer structures would progressively emerge at a certain stage from the repeated use by the signers of a deliberate intent to do as much iconic as possible^10^ in order to make themselves better understood^11^. In this context, a structural bifurcation would gradually occur, opening the way, parallel to the first one, to another mode of saying, in a generic way, with no intention of being iconic (that is, with no illustrative intent). This would result in the emergence and multiplication of lexicalized units, born from the routinization (entrenchment) of transfer units having lost their illustrative scope. However, the centrality of transfer structures is not just a diachronic or historical phenomenon. Rather, it accounts for the current discursive dynamics in SLs. The dynamics is grounded on the functional complementarity of the two available modes of saying, depending on the signer’s intent: saying while showing and saying without showing.^12^ The iconicity attested in many lexical signs, where it is not, however, the mode of meaning production, is not a pure etymological remnant, doomed to disappear. Instead, this “dormant iconicity” is functionalized: it only allows the economic integration of the two main types of structures and units into SL discourse (see next section, Figure 4).

Ultimately, deafness would be the root of SL structuring, in two respects: first, from a genetic perspective, as 95% of deaf children are born from hearing parents,^13^ the scenario proposed above for the semiogenesis of SL is very likely as old as deafness itself; secondly, from a communicative perspective, the need to maximize the communicative potential of the sole modality available, the visuo-gestural modality. From then on, the Semiological Approach is grounded on a preliminary “semiology of the channel,” the latter being understood as a modality that generates constraints but also carries its own potential. For the visuo-gestural channel, that means, first, the possibility to maintain the figurative, allowing one to reproduce *as closely as possible* the universe of mental imagery, as this is the very channel by which we experience the world, but also, the dual possibility (linked to the nature and visibility of body articulators) to reflect semantic relations in spatial terms, and to fully exploit simultaneity. The Semiological Approach thus opens up an epistemological reversal, inviting us to contemplate the *forms* used in SpL similarly, removed from their habitual privileged position in general linguistics, and instead as particular reactions to *constraints* imposed by the audio-vocal channel^14^, but also having the option, in hearing communication, to employ two modalities jointly^15^. This explains the name of the Semiological Approach, which aims, therefore, to *describe all human language*. The Semiological Approach models SL as a *type* of language, because it is rooted, on the one hand, on the incidence of deafness, and on the other, on the hypothesis of a close link between linguistic structure and mental imagery (that itself stems from experiential interactions with the world)^16^. The following section is intended to support the typological contribution of our approach.

## Typological Scope of the Semiological Approach: Reference to Entities in Different Sign Languages

The examples we present in this section are drawn from two sets of data: data in three SLs collected and analyzed by Pizzuto et al. (2008) and those from a large corpus of narratives in eleven SLs presented in Sallandre (2014, 2020), Sallandre et al. (2016).

Pizzuto et al. (2008) is the first crosslinguistic SL study on doing reference in SL carried out within our theoretical framework. The corpus is made of narratives from three signers, in LIS, ASL, and LSF, elicited from two stimuli: for LIS and ASL, the story-board *Frog, where are you?* (Mayer, 1969), and, LSF, the story-board *The Horse*. This study has shown strong similarities between the three SLs, LIS, LSF and ASL. Their results show that lexical units are favored for introducing animate or inanimate referents (50%–83%), while transfer units are the preferred method for maintaining and reintroducing referents (76%–95%). More specifically, personal transfers are used mostly for animate referents while transfers of size and shape and situational transfers are preferred for inanimate referents; double transfers are used to reintroduce referents of both types but never used to introduce new referents. Finally, a small proportion of anaphoric reference is marked through manual pointing signs (3%–7%). These initial results should be compared with the more recent results in Hodge et al. (2019), within a different theoretical framework.

**FIGURE 5:**
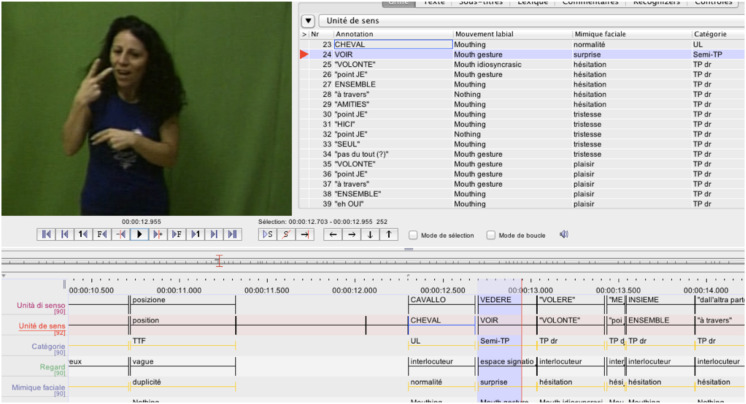
Screen capture of ELAN annotation of a story in LIS. Focus on the meaning unit VEDERE (SEE) in a semi-personal transfer structure.

Following this work, Sallandre (2014, 2020), Sallandre et al. (2016) compared reference to animates in eleven SLs, focusing in particular on how personal transfer units interact with lexical and pointing units to introduce and maintain reference. The SLs studied, as illustrated below, are LSF (French SL) LIS (Italian SL), LSR (Romanian SL), DGS (German SL), VGT (Flemish SL), PJM (Polish SL), SASL (South African SL), NS (Nihon Shuwa, Japanese SL), Libras (Brazilian SL), LCSh (Chilean SL), and LSM (Mauritian SL)^17^. These are both European and non-European SLs with diverse institutional statuses. The data were collected by us or by our colleagues in the various countries. The same narrative, *the Horse*, was produced by five deaf signers in each language; the productions were then annotated using the ELAN software (Sloetjes and Wittenburg, 2018). The same template was used to annotate all productions and is relatively synthetic, using the following fields (called *tiers* or *actors*): The tier *unit of meaning* proposes a translation of the minimal units of realization in the written SpL of the relevant country (e.g., Italian, for the LIS, cf. Figure 5) and in French, the working language of the authors. The tier *category* assigns a label to each unit (lexical unit, types of transfer, pointing, or fingerspelling). The other tiers correspond to the non-manual parameters (gaze, facial expression, mouth patterns, and body posture).^18^

The most common transfer structures are described below. Our stake is twofold. We highlight the presence of these transfer structures across all the SLs examined, whether historically related or not^19^. At the same time, we illustrate what characterizes them as structures, namely, their compositionality. We first present the three main transfer structures and then illustrate some of the most common complex regular patterns that result from a combination of these structures either with each other or with other types of units. Taking into account for each simultaneous construction all its manual and non-manual parameters, analyzed according to the signer’s intent^20^, highlights the extreme potential linguistic body partitioning.

Figure 1 demonstrates a transfer of size and shape in LSF and in Libras. This transfer unit appears in the first part of the story, in the description of the background that includes a fence. Both signers use the same “four fingers spread” handshape for the dominant hand to represent the shape of the posts of the fence. In both signers, the fingers are pointing upward, but the palms of the hands are facing in different directions (outward for the LSF, inward for the Libras). This is a personal variant, not due to the norm of either language, and it does not affect the meaning conveyed. In both cases, the eye-gaze instantiates the shape described by the dominant hand (right hand) while the facial expression shows the length and delicacy of the shape described, also suggested by the squinting of the eyes.

The two images in Figure 2 depict the same crucial moment of the story, the horse’s jump and fall. Both signers, as most other signers of this corpus, chose to express this event using the same structure, a situational transfer. The choice is probably motivated by the inherent external point of view of this transfer, which allows the signer to emphasize the harsh trajectory of the horse relative to the fence. In these two images, the meaning conveyed is very similar: in LSF (on the left), the dominant (right) hand shows the horse jumping over the fence, which is represented by the non-dominant hand; in Libras (on the right), the dominant hand also shows the horse’s jump, while the non-dominant hand figures the ground onto which the horse stumbles awkwardly. The structural similarity between the two units is obvious: meaning conveyed by the dominant hand, which represents the action of the animate referent, movement of this hand over the non-dominant hand (locative), and gaze following the action carried out by the dominant hand. The minor differences are in the proform used for the dominant hand, in LSF, a V shaped form with two saliences (two fingers stretched open), and in Libras an X shape (two folded fingers), producing two slightly different representations of the form depending on whether or not the signer intends to show, at that particular moment, the bending of the horse’s legs. The facial expressions providing aspectual value are also slightly different, expressing effort and speed in LSF and the shock of the fall in Libras.

Finally, the images in Figure 3 present a very similar personal transfer of the horse galloping, in three languages, LSF, LIS, and NS. Again, we find striking structural similarities: directing the gaze away from the addressee, the postural involvement (chest, shoulders, head), and facial expression (that of the entity), all these elements indicating an internal and embodied point of view on the scene, in contrast to the external point of view inherent to situational transfer. Depending on SLs and signers, the handshape may differ (proform “fist” as here, or proform “two outstretched fingers” or even “flat hand”), according to the aspect they intent to show, but the attitude of the signers, moving away from the “Enunciation Domain” to embody the protagonist of the utterance, is extremely similar (for a definition of the term “Enunciation Domain,” see the next section).

After having illustrated the three main transfer structures, let us move on to those that combine, simultaneously, either another transfer or a lexical or other unit. These structures, which will be outlined below, exhibit higher semantic density and more referents simultaneously present in the utterance. However, the role of the gaze is constant: in all these personal transfers, it is to represent the state of mind of the character(s) embodied as protagonists of the utterance (see section “Enunciation and Deixis-Anaphora: Key Role of Eye-Gaze”).

**FIGURE 6:**
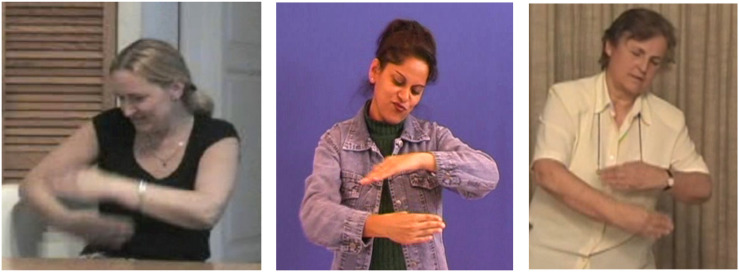
Double transfer “the cow bandages the horse’s leg” in PJM, LSM, and LCSh.

We begin by what we call “double transfer” which is the simultaneous combination of a personal transfer and a situational transfer. Double transfer allows one to simultaneously express multiple perspectives (e.g., that of an agent and of a patient or that of a locative and that of an agent). Figure 6 demonstrates an example of a double transfer in three SLs, PJM, LSM, and LCSh. This transfer is produced at the end of the Horse story, and its activation status is a simultaneous reintroduction of the two main protagonists of the narrative. It can be translated by the utterance “the cow bandages the horse’s leg.” This construction structurally combines a situational transfer locative (the horse’s leg, represented by the non-dominant hand) and a personal transfer (the cow bandaging the leg, represented by the whole body, except for the non-dominant hand). Thus, two animated referents are simultaneously present in the utterance. In all images, the parameters are the same, with the gaze oriented toward the horse’s leg, which was previously introduced into the narrative, i.e., bottom right for the signers in PJM, and bottom left for the LSM and LCSh.

**FIGURE 7:**
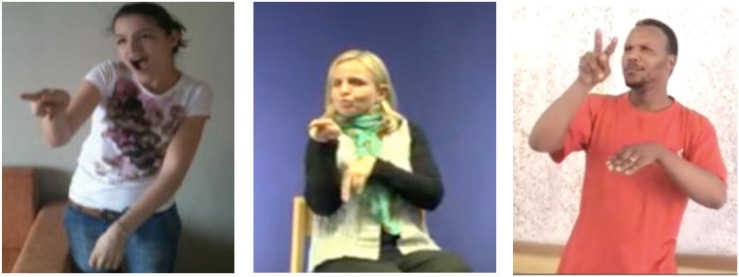
Semi-personal transfer SEE, in LSR, VGT, and SASL.

The frequent integration of elements classically associated with the non-illustrative intent (e.g., a lexical unit) into a structure depending on the illustrative intent (e.g., a personal transfer) is analyzed in the Semiological Approach as a specific type of transfer structure (e.g., a semi-personal transfer, Figures 5, 7). This type of highly frequent pattern (such as these structures) employs simultaneous constructions (i.e., units) carrying multiple references. It is consequently difficult to compare with SpL, including when multimodality is considered.

The examples in Figure 7 illustrate a semi-personal transfer in LSR, VGT, and SASL, produced at different moments in the narrative. As it is the case in our previous example in LIS (Figure 5), this structure is defined by the simultaneous association of a personal transfer, here the character of the horse, and a lexical unit that specifies the action of the transferred entity. Here, while the body of the signers (including their facial expression and gaze) depicts the horse being surprised, the action of the dominant hand indicates that the horse “looks” at the cow. This is the lexical unit SEE that is used. A similar example from Auslan is presented in Hodge et al. (2019, Figure 4, unit 5). Thus, the personal semi-transfer is found in all the SLs examined (as does the semi-double transfer described below).

These corpora were assembled to examine one of the typological hypotheses underlying the Semiological Approach, namely, the existence of various transfer structures across SLs. Beyond certain variations between signers and between SL, our analyses confirm the existence of multiple structural similarities across the SLs examined. They also confirm the richness of the transfer constructions in each SL, as evidenced by the existence of double transfers or personal transfers with reported discourse^21^. Such outcomes, we must insist, require taking into account at any moment the functions performed by all the parameters, manual and non-manual, and particularly the gaze.

In the last part of this article, we will focus on the importance of gaze behavior (the behavior of the signer’s gaze but also, crucially, that of the addressee’s) for the functioning of reference in SL. We believe, however, that only an “enunciative” approach can provide an appropriate account thereof. The Semiological Approach, which has taken a functionalist perspective from the outset, also found significant resonance in the principles of Langacker’s (1987, 1991) Cognitive Grammar, and in Lakoff’s thinking (e.g., Lakoff and Johnson, 1980/1995). However, as stated in the introduction, an important aspect that distinguishes our conception from others which also align with a cognitive–functionalist approach is the adoption, from the outset, of the perspective of the *théories de l’énonciation*, a European approach that developed notably in France over the 20th century, its proponents dominating the field in the 1960s–1980s. This approach and its close links with deixis–anaphora are the focus of the following section. We highlight its specific relevance to SL, provided that the role of the gaze in these languages is fully taken into account.

## Enunciation and Deixis–Anaphora: Key Role of Eye-Gaze

The term “enunciation theories” refers to a set of very diverse approaches which have in common that they have in-depth questioned the abstract notion of “language” (“la langue”) posed by Saussure (as opposed to “la parole”). However, as Liddle (see Culioli, 1995) and Fuchs (2008), among some others, point out, while there are important points of intersection with Cognitive Grammar, these contributions have remained largely unknown in the United States. It is not possible to reconstruct here the historical roots of the notion of “enunciation,” nor the specific contributions of its main representatives. With regard to the Semiological Approach, the key references are to be found in Jakobson (1957) and his concept of “shifter”, in Benveniste (1970) and, concerning more particularly the concept of enunciator/co-enunciator, in Culioli (see note 24).

It is indeed from the concept of “shifter” masterfully developed by Jakobson (1957) for the study of the verbal forms of Russian that Benveniste elaborates his “enunciation theory.” He shows that every utterance (“énoncé”) necessarily contains a set of terms (“indices”) whose specificity lies in the fact that they can only be defined by reference to what made it possible to produce the utterance itself, which he calls its “enunciation” (“énonciation”). These are the 1^st^- and 2^nd^-person pronouns that refer to the two interlocutors in the act of enunciation, “deictics” such as “here,” “now,” which refer to its place and time, the verbal tenses (the present tense, which designates a period of time as that of the enunciation), the way speakers embed their own personal assessment of their messages within them (the modalities), etc. Therefore, “Enunciation is this coming into service of language that is created by an individual instance of use.”^22^ (Benveniste, 1974, 80), the conditions of this activation being inscribed in the very system of language, through what is described as “the formal apparatus of enunciation” (Benveniste, 1970). Consequently, far from being a neutral and objective system, language (“la langue”) contains “indices” that are to be considered as the very basis for constructing referential values.

By this very fact, every utterance carrying within it traces of its enunciation, it should be analyzed by taking into account two “layers,” referred to as the “Enunciation Domain” (“plan de l’énonciation”) and the “Utterance Domain” (“plan de l’énoncé”). The first links and *linguistically* co-determines the speaker and the addressee: the very act of enunciation establishes them simultaneously (and reversibly) as 1^st^ and 2^nd^ person. The Utterance Domain is internal to the discourse being produced: it links the protagonists of the uttered process^23^. A key point is that the Enunciation Domain cannot be reduced to what is commonly referred to as “the utterance context,” i.e., the context understood as the physical environment and all the actual circumstances in which an utterance is produced. The absolute, actual physical coordinates of the interlocutors are not relevant from an enunciative perspective. Personal shifters express the necessarily mutual co-determination of the two “co-enunciators”^24^, and by contrast, that of the non-person (i.e., 3^rd^ person). Pizzuto (2007), summarizing Benveniste’s thought, underlines this “ineradicable subjectivity” introduced into language through the relationship between interlocutors (co-enunciators) established by the act of uttering (enunciation) and its necessarily universal nature.

Let us now recall that the issue of grammatical person marking was debated in SL linguistics very early on. As mentioned above, the long-dominant analysis identified in ASL (and later in other SLs) three personal pronouns, in 1^st^, 2^nd^, and 3^rd^ person, formally characterized, for the 1^st^ person by an index finger pointing toward the signer’s chest, for the 2^nd^ and 3^rd^ person by an index finger pointing respectively toward the addressee (2^nd^) or to the addressee’s right (3^rd^). The first to challenge this analysis was Meier (1990). Arguing that the interlocutor can alternately be one or the other of the participants (other than the signer) in an exchange, Meier points out that for both the 2^nd^ and the 3^rd^ person, the direction of pointing can be infinitely variable. This variation, he argues, poses a problem for a formal specification of these pronouns. He therefore proposes that ASL presents only a binary grammatical opposition, between 1^st^ person and non-1^st^ person. The debate also focused on the possibility of a formal analysis for the marking of person/arguments of the verb in directional verbs/agreement verbs. Recently revived, the discussion therefore focuses on the non-listable (non-morphemic) character of spatial points (loci) that can be created for doing reference. As mentioned above, Liddell (2003) provides the same analysis for both pointing signs and what he renames “indicating verbs”: the theoretically unlimited variation of their direction indicates that they are gestural (i.e., according to him, non-symbolic) in this respect. Liddell thus joins Meier’s position via another way: since manual pointing is assumed to be what formally marks the grammatical person, the unlimited variation of the actual location of the interlocutors would block the possibility of a formal distinction between the 2^nd^ and 3^rd^ person. However, Liddell goes further: he assimilates SL pointing signs to ostensive pointing gestures that can be found in SpL coverbal gesturing. In his words, “the directionality [of pointing signs] is an explicit instruction telling the addressee how to map the pronoun’s semantic pole. *The addressee needs only to follow the directionality of the pronoun*, which will lead to the appropriate entity.” (Liddell, 2003, 91, emphasis added).

Our analysis is very different. Adopting an enunciative perspective from the outset, Cuxac was particularly attentive to the gaze behavior of both interlocutors. He thus noticed that what specifies the addressee’s gaze in SL is on the contrary its fixity (Cuxac, 2000, 217)^25^ :

“Anyone who has had the chance to observe signed communication cannot be but struck by the immobility that characterizes the receiver of the message: his/her body and face remain still (except for micro oscillations of the head that play a phatic function). What is most striking, however, is *the stillness of [the addressee’s] gaze*. In order to capture the linguistic information provided by the signer’s gaze and facial mimicry, the addressee maintains his/her gaze constantly focused (with respect to central vision) on the area around [the signer’s] eyes. Most notably, *the addressee’s gaze is never directed (in foveal vision) on the gestures that are produced, and it does never follow the movements of the signer’s hands*”

This observation and its consequences are of crucial importance, as the fixity of the addressee’s gaze attests (contra Liddell) to the deep difference in nature between the pointing sign in SL and the ostensive pointing in coverbal gesture. In parallel, Cuxac points out this other seemingly trivial fact that, in these visual face-to-face languages, no communication can take place without shared gaze. Now, what defines 1^st^ and 2^nd^ person as such in SL is this interlocked gaze which is also the very condition for the establishment of an act of enunciation in these languages. Indeed, according to the Semiological Approach, the *primary* means through which signers encode person reference distinctions is not pointing signs but, precisely, eye-gaze. These gaze patterns can be combined, for the 1^st^ person, with a self-pointing and for the 2^nd^ with a pointing toward the one being looked at (co-enunciator). However, as noted in the literature, these pointing signs, which have rather an emphasis value, are often optional. The 3^rd^ person is, as opposed to the 1^st^ and 2^nd^ person, what is pointed at by the signer without being looked at (very literally the “non-person”). In an enunciative perspective, what is thus relevant is not pointing signs *per se* (nor a fortiori their actual direction) but their coupling/decoupling with gaze. This coupling/decoupling constitutes the basis of the distinction between the 1^st^ and 2^nd^ person and between them and the non-person, this operating in the two “Enunciation” and “Utterance” domains. The other salient feature is indeed, once the co-enunciators’ gazes are “interlocked,” thereby determining the Enunciation Domain, the extreme mobility of the signer’s gaze, as opposed to the fixity of his/her addressee’s: “(…) the signer’s gaze is extremely mobile, and meaningfully redirected toward the points in space that mark deictic-anaphoric reference in the ‘third-person domain’ in discourse” (Pizzuto, 2007, 19).

**FIGURE 8:**
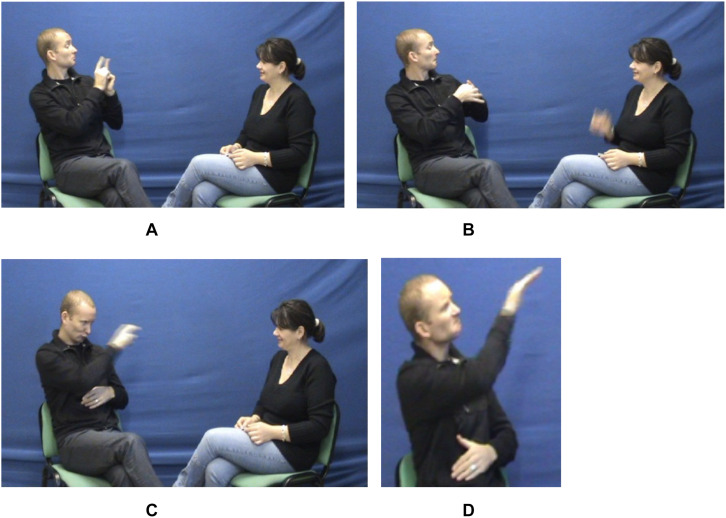
Interweaving between both types of iconicity and space. **(A)** Lexical unit MEET, **(B)** lexical unit WIFE, **(C)** lexical unit PICK UP, **(D)** lexical unit CALL.

**FIGURE 9:**
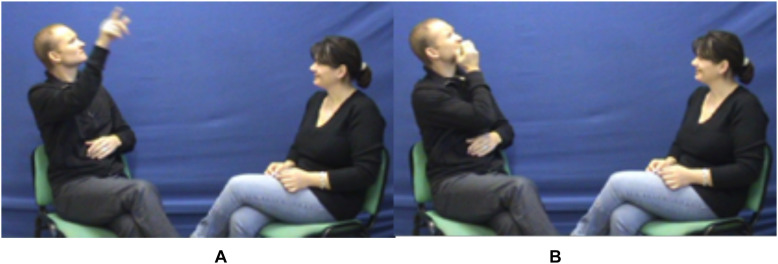
Embedding a level 2 enunciation frame of reference within the Utterance Domain. **(A)** Pointing sign outside the axis of interaction (3rd person), **(B)** lexical unit BITE ONE’S NAILS.

In fact, both prerequisite for the advent of any signed interaction and an anchor point for the personal deixis established by the very act of enunciation, the signer’s gaze is also the key operator for creating and tracking (personal, temporal, and spatial) references in SL discourse. Thus, where the signer’s intent is non-illustrative, it is his/her gaze (sometimes coupled with a manual pointing, either preceding, accompanying, or following) that activates a specific point in space (locus), prior to a lexical unit being spatialized there. The signer’s gaze alone is subsequently sufficient to reactivate the locus, thereby reactivating the associated referent. In other words, it is primarily the gaze that *diagrammatizes* space, enabling a weave of semantic (grammatical) relations between entities associated with these loci. According to us, the use of space in SL is therefore of two types: (i) a topographical or descriptive space, which is an imagistic space and characterizes reference under the illustrative intent, and (ii) a diagrammatic space, typically involved in the construction and tracking of reference outside the illustrative intent. This, however, must be complemented by taking into account, from an enunciative perspective, the opposition mentioned above between the Enunciation Domain and the Utterance Domain and the different discursive frames of reference they generate. The following example^26^ (Figures 8, 9), which combines the two modes of meaning production, will illustrate these points, beginning with the degree of complexity the imbrication of the two types of spaces (imagistic and diagrammatic spaces) can achieve as well as the corollary finesse of the signer’s management of his gaze. It should be pointed out that the sequences of images do not claim to represent the entire discourse, but rather selected moments.

The signer in this sequence is the director of an association teaching LSF to hearing people. In the example, he is explaining to the addressee the origin of his sign name. To do that, the signer refers back to the time he met his wife. He looks to some point on his left and thus activates a locus on which he immediately places the lexical unit MEET (Figure 8A) and then the lexical unit HUSBAND-WIFE (Figure 8B), which means “I meet [my] wife.” Henceforth, this locus stands for the “wife” entity, following the diagrammatic logic mentioned above. Then follows a short sequence where the signer explains that at that point, his wife worked in a school for deaf children and that, in order to save money, he picked her up at work (Figure 8C). Looking at the pre-activated locus to his left, the signer produces the directional unit PICK-UP and orients it toward the “wife” locus with a motion of his chest (“I (therefore) was the one who picked her up”). In the immediately following sequence (not shown here), the locus “wife” is repeatedly reactivated and used as such. The signer explains that he was on this occasion regularly observed by the schoolchildren, and in particular by one of them. Therein lies the interesting point: switching to the other mode of meaning production (saying while showing) by breaking up the shared gaze, the signer continues his story by embodying himself in this child. To do this, he uses a personal transfer, enabling him to incarnate an entity distinct from himself (a 3rd p.) whose actions, thoughts, etc., he can show. The shift of his gaze away from his addressee’s, which is typical of transfers, signals that the signer is no longer the enunciator; from that point on, his gaze is the gaze of the child he is transferred in. Following the imagistic logic which specifies the mode of meaning production within transfers, the signer (who became the “child” entity) articulates the directional lexical unit CALL through an orientation (reflected by the gaze) toward a point *higher* on his left (Figure 8D), meaning “the child calls my wife.” What is noteworthy is that, respecting the logic of the previously elaborated and still active diagrammatic space, the signer positions his locus to his left, but he does so while simultaneously conforming to the logic of the imagistic space opened by the personal transfer: having become the “child” entity, he locates the “woman” entity higher up according to this latter logic. The two types of iconicity and the two corresponding types of space are thus combined in a way that is as economical as it is rigorous: the diagrammatic space of the relations between the actors of the utterance and the imagistic space opened by the personal transfer (space of the transferred entity).

Let us now illustrate, with the following part of the same sequence (Figures 9A,B), what we mean by “enunciative space” or “enunciative frame of reference” (enunciation space and utterance space) and the complexity of the constraints the signer must respect in managing his gaze within these intertwined spaces.

Having called the signer’s wife (see above Figure 8D), the entity “child” engages in a dialogue with her (i.e., the entity represented by the locus “wife”). Becoming therefore a level 2 enunciator, the child–signer entity determines by this very fact the wife entity as co-enunciator (2^nd^ person) by looking at it (signer’s gaze on the “wife” locus). In that level 2 Enunciation Domain thus opened within the first level utterance (reported speech), the “child”–enunciator produces a pointing sign (also in height, Figure 9A) outside the axis of interaction *while maintaining his gaze (raised) on his co-enunciator*, that is on the locus–wife (marking of the 3^rd^ person: “he”) and then he produces the lexical unit BITE ONE’S NAILS (Figure 9B). The reported utterance can be translated as follows: “He (= level 1 enunciator-signer, now a 3^rd^ person) bites his nails.”^27^ What is noteworthy is the reiteration within space of the level 2 enunciation frame of reference of the principle described above for the formal marking of the grammatical person by the dynamics of the shared gaze (1^st^/2^nd^ person) and its decoupling from the direction of manual pointing (3^rd^ person).

The extreme logic and precision with which the signer manage the deictic functions of his gaze thus makes the whole discussion about the “real” coordinates of the interlocutors and the alleged infinite variability of the loci somewhat pointless. On the contrary, it seems to us that an enunciative analysis such as the one we are proposing, based on the key role of the gaze in these languages of the visual and face-to-face that are, in essence, SLs, is able to give the simplest account of their own linguistic economy.

Let us come now to a key opposition in the gaze behavior, which is to signal the signer’s semiological intent. As we mentioned earlier, while, outside the illustrative intent, the signer’s gaze creates deixis (activating an/or reactivating loci in the signing space), it is yet primarily used to maintain eye contact with the addressee, particularly during the production of lexical units. At the opposite, the intent to say while showing (illustrative intent) requires the signer’s gaze to be detached from the addressee, thereby signaling the temporary removal of the signer as enunciator. What distinguishes indeed the illustrative intent, and is therefore shared by the three main transfer types, is the prolonged break of eye contact between the signer and the addressee. By breaking the shared gaze, the signer literally erases him/herself from the Enunciation Domain. In personal transfer, the signer actually disappears as enunciator and embodies an entity referred to in the Utterance Domain, his/her gaze becoming that of the transferred entity (Figure 3). In situational transfer, the signer’s gaze follows the movement of the entity being referred to by the dominant hand (Figure 2). In size and shape transfer, the signer’s gaze accompanies the display of the shape (Figure 1). Therefore, the signer’s gaze is a crucial clue to his/her semiological intent.

**FIGURE 10:**
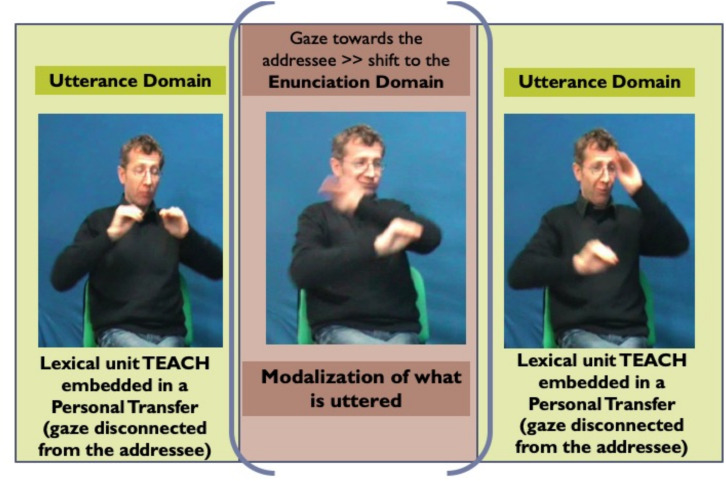
Screenshot of the semi-personal transfer “TEACH awkwardly,” from Garcia and Sallandre (2014, 330).

However, the analysis of gaze direction and the associated function requires a rather broad discursive context. An example will illustrate this point, while allowing us to refine our presentation of the roles of the gaze. Thus, while in the midst of producing a transfer structure, the signer can briefly direct the gaze toward the addressee intentionally, as if pausing the manual production, thereby momentarily reestablishing the Enunciation Domain; in this way, now reappearing as the enunciator, the signer can comment on the utterance, through facial expressions, thus “modalizing” it. Let us see the following example, pictured in Figure 10.

In this sequence, the signer describes his career as an LSF teacher to hearing adults. He resorts to a personal transfer of himself at the beginning of his career, showing himself as the clumsy professor he was. While embodying the young teacher he used to be, he produces the lexical unit TEACH. This embedding of a lexical unit in a broader illustrative context in which the signer is using a personal transfer^28^ stems from a semi-personal transfer (see above Figures 5, 7). As we have seen, this is a very economical structure precisely because the conventional and generic information carried by the lexical unit and the information conveyed by the iconic mode of meaning production (*saying while showing*) overlap, as witnessed by the manual and non-manual multi-linearity characteristic of the illustrative intent. However, complexity is further increased by the play of gaze (and facial expression), which allows the signer to shift from the Utterance Domain (where he stands as an embodied entity) to the Enunciation Domain (where he interacts with the addressee/co-enunciator). In fact, during the personal transfer of himself as a young professor (lexical unit TEACH), teaching awkwardly (hand movement and orientation), the signer’s gaze and facial expression are alternatively: (i) those of the transferred character (himself at the time)—his gaze set on the moving hands, i.e., disconnected from the addressee, thereby signaling the transfer, and his facial expression depicting the muddled and awkward nature of the process (of teaching) (Figure 10, left and right images) and (ii) those of the signer/enunciator commenting to the addressee/co-enunciator on his teaching experience, displaying self-deprecation—his gaze set on the addressee, with a self-deprecating facial expression. Such sequences, whose complexity arises from the intertwining of lexical and transfer units, alternating between the two modes of saying, and from the interplay between the Enunciation and Utterance Domains are very characteristic of SL discourse.

Finally, acting as a rector for changing the frame of reference, the gaze is also what determines the shift from one intent to the other. In fact, it is often enough for the signer to direct his/her gaze on a lexical unit (by definition not looked at) so that, by switching to the illustrative intent, the latter shed its conventionalized nature and deploys its iconic potential, either by reactivating an original iconicity (in a lexical unit that stems from a transfer unit) or by re-motivation (reanalysis). To illustrate, in Figure 4, the gaze is initially directed toward the addressee during the production of the lexical unit TREE (unit 1); then, after a slight nod, the gaze turns toward the sign itself (unit 2); the lexical unit becomes a proform that iconically depicts the tree, and its branches, in particular (unit 2). This opens up the possibility of creating a construction around this locative proform, which is first activated by a manual pointing (unit 3) and becomes the situational transfer’s locative on which sits the bird, i.e., animated agent of the utterance (unit 4). Thus, as indicated above, the iconicity present in many lexical signs is what allows the back and forth between the two modes of saying and thus between the main types of structures, in a particularly economical way.

In the end, as a condition for the advent of a signed interaction, anchoring of the personal deixis, rector of the referential framework, marker of the signer’s intent, key operator of the diagrammatization of space (activator and re-activator of reference), vector of the modalization of the utterance, the gaze is plurifunctional in SL. This is why, we insist once again, in order to properly analyze the function that the gaze fulfils at a given point, a sufficiently broad part of the discourse must be taken into account.

## Conclusion

If we want to be able to compare crosslinguistic data on an equal basis to determine how reference operates in SL discourse, it seems to us urgent that SL linguists come to an agreement on how to segment sequences. From our perspective, namely, that of the Semiological Approach, segmentation requires an equal consideration of *all* meaning-carrying parameters, manual and non-manual alike, from a “vertical” view of the minimal unit of realization (see above, note 7, on Cuxac’s, 2013 “multi-track body matrix”). While every parameter, non-manual ones in particular, plays a role in this matrix, this role is affected by the signer’s intent, saying without showing, on the one hand, and saying while showing, on the other hand. Intent is defined by the gaze, whose role in this capacity is crucial.

We have stressed the multiple functions of the gaze and the importance of taking into account two key observations, from an enunciative perspective. The first observation is that, in these visual languages, where communication is by nature face-to-face, it is the shared eye-gaze that anchors deixis. The second one is the fixedness of the addressee’s gaze, which maintains focused on the signer’s. This is sufficient to highlight the profound difference in nature between (linguistic) pointing signs in SL and ostensive pointing gestures that can be found in SpL coverbal gesturing (*contra* Liddell, 2003). On this basis, we have endeavored to show how the distinction between Enunciation Domain and Utterance Domain, on which “enunciation theories” are based, is able to account for the most complex discourses in SL in a particularly economical way, provided that the signer’s gaze is accurately taken into account.

Conducted in this perspective, our analyses of discourse sequences from corpora in several SLs attest to the multiple structural similarities notably with regard to transfer structures. This result strongly supports the typological hypothesis underlying the Semiological Approach, that all SLs share a substantial structural base, consisting notably of this type of structure.

Clarifying more precisely the difference in nature between the information conveyed by each of the two main types of “semiotics,” saying without showing and saying by showing, should enable us to refine our understanding of “what reference is” and how it is established in SL. The fact that SL linguistically uses these two major semiological modes of saying offers linguists who are open to the multimodality of human language the opportunity to take an innovative look at the age-old theme of deixis–anaphora and thus renews the debate.

## Data Availability Statement

Most of the data sets presented in this study are available online. The name(s) of the deposit(s) and accession number(s) can be found below:

– Corpus LS-Colin: https://cocoon.huma-num.fr/exist/crdo/ark:/87895/1.17-483699;
– Corpus Creagest: https://www.ortolang.fr/market/corpora/ortolang-000926;
– VGT Corpus: https://www.corpusvgt.ugent.be/ and more specifically https://www.corpusvgt.ugent.be/nl/videoresultaat/1010 (Figure 7).

## Ethics Statement

Ethical review and approval was not required for the study on human participants in accordance with the local legislation and institutional requirements. The patients/participants provided their written informed consent to participate in this study. Written informed consent was obtained from the individual(s) for the publication of any potentially identifiable images or data included in this article.

## Author Contributions

BG designed the manuscript, wrote sections “Introduction; Reference, Deixis and Anaphora: Background; The Semiological Approach to Sign Language; and Enunciation and Deixis-Anaphora: Key Role of Eye-Gaze.” M-AS wrote sections “Typological Scope of the Semiological Approach: Reference to Entities in Different Sign Languages; Conclusion,” selected the data in twelve SLs and transcribed the LSF videos, designed and edited the figures. Both authors contributed to the conception of the study and thoroughly analyzed and discussed the data.

## Conflict of Interest

The authors declare that the research was conducted in the absence of any commercial or financial relationships that could be construed as a potential conflict of interest.

## Footnotes

1 For a historical perspective of SL linguistics, see in particular Newport and Supalla (2000), Vermeerbergen (2006), and, for a French perspective, Garcia (2010), Garcia under review.

2 We fully agree with one of our reviewers that Liddell’s use of the term “gradient” is somewhat confusing and inconsistent with Langacker’s understanding of the term. For Langacker indeed, “gradience” is everywhere in language and, far from being opposed to “symbolic,” it is inherent to it (on this point, see in particular Occhino and Wilcox, 2017).

3 At that period, this could not have been a corpus in the modern sense of the term, i.e., *machine-readable*, as defined by Johnston (2008). Yet, given that discourse data were mainly collected in an ecological manner and transcribed frame by frame following an explicit procedure (see Cuxac, 2000; †Antinoro Pizzuto and Garcia, under review), it is as close as one can get. The important point here is that the description and theory that followed were authentically *corpus-driven* (vs. *corpus-based*, *cf.* Tognini-Bonelli, 2001).

4 For a detailed description of what these three main transfer structures are precisely made of, the reader may refer to Cuxac and Sallandre (2007) and Garcia and Sallandre (2014).

5 We return to this term later (see note 11 below).

6 See examples below (Figures 2, 3).

7 The loci are therefore not intended to reflect the actual absolute positions of the entities they represent, but rather the (person, space, time) relationships between these entities.

8 Cuxac (2013, 78) refers to these two main types of SL discourse units as “multi-track body matrix with relevant cells that are more or less filled in (eye-gaze, body posture, facial expression, manual parameters) depending on the structure achieved” [our translation; French: “(…) une matrice corporelle multipiste à cases pertinentes plus ou moins remplies (regard, posture, MF [mimique faciale], paramètres manuels) en fonction de la structure réalisée »].

9 Similarly, see the description of a micro-community SL in Senegal, Jirou-Sylla (2008).

10 This hypothesis of an “intent” to be “as iconic as possible” is a key one for the Semiological Approach, both at the diachronic and ontogenetic levels and in the synchronic use of language. As an explanatory hypothesis, it is of the same nature as, among other examples, the one that underlies Ariel’s Accessibility Theory, for which the speaker/signer would choose his/her referential expressions according to the degree of accessibility of this referent that he assumes in his interlocutor.

11 If so, the transfer structures (and their components) are what gives us the closest insight into what “iconic” means. They are indeed considered as the product of one human being’s efforts to make himself understood by another human being by making maximum use of the resources of a channel that also happens to be the very channel through which they both experience and conceptualize the world.

12 For examples of such functional complementarity, see in particular Garcia and Sallandre (2014).

13 The atypical language acquisition of deaf children from hearing families is thus, from the very beginning, a corner stone of our approach (see notably Cuxac et al., 1999).

14 We sincerely thank the reviewer who drew our attention to the astonishing similarity with the words of the great Hockett, of which we give only snippets: “The difference of dimensionality means that signages can be iconic to an extent to which [spoken] languages cannot. […], so that it is perhaps more revealing *to put the difference the other way around, as a limitation of spoken languages*. Indeed, *the dimensionality of signing is that of life itself* […]” (Hockett, 1978, 274–275, quoted by Wilcox, 1996, 184), *emphasis added*. In the same vein, we fully subscribe to the model recently developed by Occhino (2016, 2017); she writes for instance: “By beginning with signed languages, and expanding our analysis to spoken languages (*the opposite of the typical direction of linguistic theory building*), we gain keen insight into the nature of schematization and emergence of structure, *which is obfuscated by the opaqueness of the articulatory mechanism of spoken languages*” (Occhino, 2017, 94, *emphasis added*).

15 Herein lies the explanation to the differences between coverbal gesture in hearing contexts and SL transfer structures, beyond any similarity. On this point, see Cuxac (2008).

16 That is why we consider iconicity not only as a “pervasive aspect” in SL but as their “organizing principle.”

17 We use the acronyms validated by the deaf communities involved.

18 While tiers for manual and non-manual parameters are almost always present in SL literature, the tier for *category* (tag) seems to be original: it can be filled as the result of parametric field values (manual and non-manual). For example, if a unit of meaning incorporates some aspectual facial expression, a gaze directed at the dominant hand, which represents the moving agent while the non-dominant hand represents a stable entity, then we have a situational transfer, which will therefore be annotated as such in the category tier.

19 We have chosen SLs that are relatively close, historically and geographically, like LSF and LIS, as well as unrelated SLs, such as LSF and NS.

20 Non-manual parameters fulfill various functions depending on the intent used. Given space limitations, we refer the reader to Cuxac and Sallandre (2007) for further detail.

21 This last type of transfer is not presented here: we refer the reader to the detailed example below, Figure 9.

22 French: “L’énonciation est cette mise en fonctionnement de la langue par un acte individuel d’utilisation.” [our translation]

23 The distinction between these two domains/layers is of particular importance. Of course, we cannot pretend to account here for the specificities of the Enunciation Theories with regard, in particular, to Langacker’s Cognitive Grammar, and we refer the reader to the rare studies on this point (see, in particular, Liddle’s analysis of the proximities and differences between Culioli and Langacker, in Culioli, 1995). However, we will say that if, from an enunciative perspective language is similarly considered as a tool for conceptualizing the world, it is first and foremost a tool for interaction.

24 Here lies what has been analyzed by several authors (e.g., De Voguë, 1992) and by Culioli himself as the major source of divergence between Benveniste and Culioli; namely the clearer and crucial difference established by Culioli between the “speaker”/the “addressee” (who refer to human beings in actual contexts of utterance but not to abstract coordinates in the “Enunciation Domain”) and what he theorized by contrast as “enunciator” and “co-enunciator.” The Semiological Approach is on this aspect closer to Culioli.

25 Translation from Pizzuto (2007, 15, note 4); emphasis added.

26 The examples in Figures 8–10 are taken from another corpus, the Creagest corpus, consisting of dialogues between deaf adults (Garcia et al., 2015).

27 The outcome of the sequence is to explain that it is this sign of “The one who bites his nails” that has become the signer’s sign name.

28 As we have seen, the micro-sequence described here is part of a larger sequence with a clear illustrative intent both before and after the chosen example.

## References

[B1] Ahlgren I. (1990). “Deictic pronouns in Swedish and Swedish Sign Language,” in *Theoretical Issues in Syntactic Theory*, eds Fischer S. D. Siple P., (Chicago: University of Chicago Press), 167–174.

[B2] Apothéloz D. (1995). *Rôle et Fonctionnement de L’anaphore Dans La Dynamique Textuelle.* Genève: Droz.

[B3] Apothéloz D. Pekarek Doehler S. (2003). Nouvelles perspectives sur la référence: des approches informationnelles aux approches interactionnelles. *Verbum* 25 109–135.

[B4] Ariel M. (1988). Referring and accessibility. *J. Linguist.* 24 65–87. 10.1017/s0022226700011567

[B5] Ariel M. (1990). *Accessing Noun-Phrase Antecedents.* London: Routledge.

[B6] Armstrong D. Stokoe W. C. Wilcox S. E. (1995). *Gesture and the Nature of Language.* Cambridge, MA: Cambridge University Press.

[B7] Benveniste E. (1966). *Problèmes de Linguistique Générale.* Paris: Gallimard.

[B8] Benveniste E. (1970). L’appareil formel de l’énonciation. *Langages* 5 12–18. 10.3406/lgge.1970.2572

[B9] Benveniste E. (1974). *Problèmes de Linguistique Générale.* Paris: Gallimard.

[B10] Brennan M. (1992). “The visual world of British sign language. An introduction,” in *Dictionary of British Sign Language/English*, ed. Brien D., (London: Faber and Faber), 1–118. 10.1017/cbo9781139167048.002

[B11] Clark H. (1996). *Using Language.* Cambridge, MA: Cambridge University Press.

[B12] Collins-Ahlgren M. (1990). “Spatial-locative predicates in Thai Sign Language,” in *Sign language research: Theoretical Issues*, ed. Lucas C., (Washington, DC: Gallaudet University Press), 103–117.

[B13] Cormier K. Smith S. Zwets M. (2013). Framing constructed action in British Sign Language narratives. *J. Pragm.* 55 119–139. 10.1016/j.pragma.2013.06.002

[B14] Cornish F. (1990). “Anaphore pragmatique, référence, et modèles du discours,” in *L’anaphore et Ses Domaines*, eds Kleiber G. Tyvaert J.-E., (Metz: Centre d’Analyse Syntaxique), 81–96.

[B15] Cornish F. (2014). “Indexicals and context: context-bound pre-requisite(s), ongoing processing and aftermaths of the discourse referring act,” in *Nouvelles Perspectives sur L’anaphore: Points de Vue Linguistique, Psycholinguistique et Acquisitionnel*, eds Fossard M. Béguelin M.-J., (Berlin: Peter Lang), 5–33.

[B16] Croft W. (2003). “Agreement as anaphora, anaphora as coreference,” in *Languages Across Boundaries: Studies in Memory of Anna Siewerska*, eds Bakker D. Haspelmath Berlin M., (Berlin: De Gruyter Mouton), 99–117.

[B17] Culioli A. (1990). *Pour une Linguistique de L’énonciation.* Paris: Ophrys.

[B18] Culioli A. (1995). “Cognition and representation in linguistic theory,” in *Texts Selected*, ed. Liddle M., (Amsterdam: John Benjamins).

[B19] Cuxac C. (1985). “Esquisse d’une typologie des langues des signes,” in *Autour de La Langue Des Signes*, ed. Cuxac C., (Paris: Université René Descartes), 35–60.

[B20] Cuxac C. (1993). Iconicité des langues des signes. *Faits de Langue.* 15 47–56. 10.3406/flang.1993.1034

[B21] Cuxac C. (1999). “The expression of spatial relations and the spatialization of semantic relations in french sign language,” in *Language Diversity and Cognitive Representations*, eds Fuchs C. Robert S., (Amsterdam: John Benjamins), 123–142. 10.1075/hcp.3.11cux

[B22] Cuxac C. (2000). *La Langue des Signes Française (LSF). Les voies de l’iconicité.* Paris: Ophrys.

[B23] Cuxac C. (2008). Langue des Signes et gestuelle co-verbale : pour un programme commun de recherches. *Les Cahiers de Linguistique Analogique* 5 181–228.

[B24] Cuxac C. (2013). Langues des signes : une modélisation sémiologique. *La Nouvelle Rev. l’adaptation et de la Scolarisation* 64 65–80. 10.3917/nras.064.0065 18052372

[B25] Cuxac C. Antinoro-Pizzuto E. (2010). Emergence, norme et variation dans les langues des signes : vers une redéfinition notionnelle. *Lang. Soc.* 131 37–53. 10.3917/ls.131.0037 18052372

[B26] Cuxac C. Braffort A. Choisier A. Collet C. Dalle P. Fusellier I. (2002). Available online at: https://cocoon.huma-num.fr/exist/crdo/ark:/87895/1.17-483699 (accessed September 1, 2020).

[B27] Cuxac C. Fusellier-Souza I. Sallandre M.-A. (1999). Iconicité des langues des signes et catégorisations. *Sémiotiques* 16 143–166.

[B28] Cuxac C. Sallandre M.-A. (2007). “Iconicity and arbitrariness, in French Sign Language: Highly Iconic Structures, degenerated iconicity and diagrammatic iconicity,” in *Verbal and Signed Languages. Comparing Structures, Constructs and Methodologies*, eds Pizzuto E. Pietrandrea P. Simone R., (Berlin: Mouton de Gruyter), 13–33. 10.1075/ill.12.04tur

[B29] De Voguë S. (1992). Culioli après Benveniste : énonciation, langage, intégration. *Linx* 26 77–108. 10.3406/linx.1992.1238

[B30] Deuchar M. (1984). *British Sign Language.* London: Routledge and Kegan Paul.

[B31] Ducrot O. (1972). *Dire et ne pas dire.* Paris: Hermann.

[B32] Ducrot O. (1980). *Les Mots du discours.* Paris: Minuit.

[B33] Enfield N. J. (2009). *The Anatomy of Meaning: Speech, Gesture, and Composite Utterances.* Cambridge, MA: Cambridge University Press.

[B34] Enfield N. J. (2013). “Reference in conversation,” in *The Handbook of Conversation Analysis*, eds Sidnell J. Stivers T., (Malden, MA: Wiley-Blackwell), 433–454. 10.1002/9781118325001.ch21

[B35] Engberg-Pedersen E. (1993). *Space in Danish Sign Language: The semantics and morphosyntax of the use of space in a visual language.* Hamburg: Signum Press.

[B36] Engberg-Pedersen E. (2003). “How composite is a fall? Adults’ and children’s descriptions of different types of falls in danish sign language,” in *Perspectives on Classifier Constructions in Sign Languages*, ed. Emmorey K., (Mahwah, NJ: Lawrence Erlbaum Associates), 311–332.

[B37] Fauconnier G. Turner M. (1996). “Blending as a central process of grammar,” in *Conceptual Structure, Discourse and Language*, ed. Goldberg A. E., (Stanford: Center for the Study of Language and Information Publications), 113–130.

[B38] Fenlon J. Cormier K. Brentari D. (2018). “The phonology of sign languages,” in *The Routledge Handbook of Phonological Theory*, eds Hannahs S. J. Bosch A., (New York, NY: Routledge), 453–475.

[B39] Ferrara L. Hodge G. (2018). Language as description, indication, and depiction. *Front. Psychol.* 9:716. 10.3389/fpsyg.2018.00716 29875712 PMC5974176

[B40] Fillmore C. (1975). *Lectures on Deixis.* Santa Cruz: Indiana University Linguistics Club.

[B41] Friedman L. (1975). On the semantics of space, time and person in American Sign Language. *Language* 51 940–961. 10.2307/412702

[B42] Friedman L. (1977). *On the other hand: New Perspectives on American Sign Language.* New York, NY: Academic Press.

[B43] Frishberg N. (1975). Arbitrariness and iconicity: historical change in American Sign Language. *Language* 51 676–710.

[B44] Fuchs C. (2008). “Linguistique française et cognition,” in *Congrès Mondial de Linguistique Française 021.* 10.1051/cmlf08340

[B45] Fusellier-Souza I. (2004). *Sémiogenèse des Langues des Signes : Étude de Langues des Signes Primaires (LSP) Pratiquées Par des Sourds Brésiliens.* Ph. D. thesis, University of Paris, Saint-Denis.

[B46] Fusellier-Souza I. (2006). Emergence and development of Signed Languages: from diachronic ontogenesis to diachronic phylogenesis. *Sign Lang. Stud.* 7-1 30–56.

[B47] Fusellier-Souza I. (2012). “Multiple perspectives on the emergence and development of human language: B. Comrie, C. Perdue and D. Slobin,” in *Comparative perspectives on language acquisition: a tribute to Clive Perdue*, eds Watorek M. Benazzo S. Hickmann M., (Bristol: Multilingual Matters), 223–244. 10.21832/9781847696045-014

[B48] Garcia B. (2010). *Sourds, Surdité, Langue(s) Des Signes et Épistémologie des Sciences du Langage. Problématiques de la Scripturisation et Modélisation des Bas Niveaux en Langue des Signes Française (LSF).* Habilitation thesis, University of Paris, Saint-Denis.

[B49] Garcia B. L’Huillier M.-T. Vincent C. (2015). *Corpus Creagest. Dialogue en LSF Entre Adultes Sourds.* Available online at: https://www.ortolang.fr/market/corpora/ortolang-000926 (accessed September 1, 2020).

[B50] Garcia B. Sallandre M.-A. (2014). “Reference resolution in French Sign Language (LSF),” in *Crosslinguistic Studies on Noun Phrase Structure and Reference*, eds Cabredo Hofherr P. Zribi-Hertz A., (Boston: Brill), 316–364. 10.1163/9789004261440_012

[B51] Garcia B. Sallandre M.-A. L’Huillier M.-T. (2018). Impersonal human reference in French Sign Language (LSF). *Sign Lang. Linguist.* 21 308–334.

[B52] Goldin-Meadow S. (1991). “When does gesture become language? A study of gesture used as a primary communication system by deaf children of hearing parents,” in *Tools, Language and Cognition in Human Evolution*, eds Gibson K. R. Ingold T., (Cambridge, MA: Cambridge University Press), 63–85.

[B53] Goldin-Meadow S. (2003). *The Resilience of Language.* New York, NY: Psychology Press.

[B54] Goldin-Meadow S. Brentari D. (2017). Gesture, sign, and language: the coming of age of sign language and gesture studies. *Cambridge Core Behav. Brain Sci.* 40:e46. 10.1017/S0140525X1600039X 26434499 PMC4821822

[B55] Hockett C. F. (1978). In search of Jove’s brow. *Am. Speech* 53 243–313. 10.2307/455140

[B56] Hodge G. Ferrara L. N. Anible B. D. (2019). The semiotic diversity of doing reference in a deaf signed language. *J. Pragm.* 143 33–53. 10.1016/j.pragma.2019.01.025

[B57] Jacob S. (2007). *Le Mouvement Référentiel Dans Des Narrations Enfantines en LSF : Conduite Descriptive Selon Une Trajectoire Développementale.* Ph.D. thesis, University of Paris, Saint-Denis.

[B58] Jakobson R. (1957). *Shifters, Verbal Categories and the Russian Verb.* Cambridge, MA: Harvard University Press.

[B59] Jirou-Sylla G. (2008). Description d’une langue des signes informelle micro-communautaire. Analyse lexicale du parler gestuel de Mbour (Senegal). *Cahiers Linguist. Analog.* 5 135–180.

[B60] Johnston T. (1991). Transcription and glossing of sign language texts: examples from Auslan (Australian sign language). *Int. J. Sign Linguist.* 2:1.

[B61] Johnston T. (2008). “Corpus linguistics and signed languages: no lemmata, no corpus,” in *Proceedings of The 3rd Workshop on the Representation and Processing of Sign Languages, (LREC 2008)*, Marrakech, 82–87.

[B62] Johnston T. (2012). Lexical frequency in signed languages. *J. Deaf Stud. Deaf Educ.* 17 163–193. 21841168 10.1093/deafed/enr036

[B63] Johnston T. (2019). *Auslan Corpus Annotation Guidelines [August 2019 Version].* Sydney: Macquarie University.

[B64] Johnston T. Schembri A. (1999). On defining lexeme in a sign language. *Sign Lang. Linguist.* 2 115–185. 10.1075/sll.2.2.03joh

[B65] Johnston T. Schembri A. (2007). *Australian Sign Language (Auslan): An Introduction to Sign Language Linguistics.* Cambridge, MA: Cambridge University Press.

[B66] Jouison P. (1986/1995). *Écrits sur la Langue des Signes Française. Edition prepared by B. Garcia.* Paris: L’Harmattan.

[B67] Kegl J. (1976). *Pronominalization in American Sign Language.* Master’s thesis, MIT, Cambridge, MA.

[B68] Kegl J. Wilbur R. (1976). “When does structure stop and style begin? Syntax, morphology, and phonology vs stylistic variation in American Sign Language,” in *Papers from the Twelfth Regional Meeting*, eds Mufwene C. Steever S., (Chicago: University of Chicago Press), 376–396.

[B69] Kendon A. (1988). “How gestures can become like words,” in *Cross-Cultural Perspectives in Nonverbal Communication*, ed. Poyatos F., (Toronto: Hogrefe), 131–141.

[B70] Lakoff G. Johnson M. L. (1980/1995). *Metaphors We Live By.* Chicago, IL: University of Chicago Press.

[B71] Langacker R. W. (1987). *Foundations of Cognitive Grammar: Volume I, Theoretical Foundations.* Stanford: Stanford University Press.

[B72] Langacker R. W. (1991). *Foundations of Cognitive Grammar. Volume II, Descriptive Application.* Stanford: Stanford University Press.

[B73] Levinson S. C. (1983). *Pragmatics.* Cambridge, MA: Cambridge University Press.

[B74] Liddell S. K. (1995). “Real, surrogate, and token space: grammatical consequences in ASL,” in *Language, Gesture and Space*, eds Emmorey K. Reilly J., (Hillsdale, NJ: Lawrence Erlbaum Associates), 19–41.

[B75] Liddell S. K. (2003). *Grammar, Gesture, and Meaning in American Sign Language.* Cambridge, MA: Cambridge University Press.

[B76] Lillo-Martin D. Klima E. S. (1990). “Pointing out differences: ASL pronouns in syntactic theory,” in *Theoretical Issues in Syntactic Theory*, eds Fischer S. D. Siple P., (Chicago, IL: University of Chicago Press), 191–210.

[B77] Lombardi Vallauri E. (2007). “The deep relation between deixis and anaphora,” in *Verbal and Signed Languages. Comparing Structures, Constructs and Methodologies*, eds Pizzuto E. Pietrandrea P. Simone R., (Berlin: Mouton de Gruyter), 309–338.

[B78] Lyons J. (1977). *Semantics. Volume II.* Cambridge, MA: Cambridge University Press.

[B79] Mandel M. (1977). “Iconic devices in american sign language,” in *On the Other Hand*, ed. Friedman L., (New York, NY: Academic Press), 57–107.

[B80] Martinod E. (2019). *Approche Typologique Des Composants Minimaux Porteurs De Sens Dans Plusieurs Langues Des Signes (LS) Se Situant à Divers Degrés De Communautarisation.* Ph.D. thesis, University of Paris, Saint-Denis.

[B81] Martinod E. Garcia B. Fusellier I. (2020). “Meaningful handshapes in the emerging sign languages on marajó island (Brazil) in a typological perspective,” in *Emerging Sign Languages of the Americas*, eds Le Guen O. Safar J. Coppola M., (Berlin: Mouton de Gruyter).

[B82] Mayer M. (1969). *Frog, Where Are You?.* New York, NY: Dial Press.

[B83] McNeill D. (1992). *Hand and Mind: What Gestures Reveal about Thought.* Chicago, IL: Chicago University Press.

[B84] Meier R. P. (1990). “Person deixis in American Sign Language,” in *Theoretical Issues in Syntactic Theory*, eds Fischer S. D. Siple P., (Chicago: University of Chicago Press), 175–190.

[B85] Metzger M. (1995). “Constructed dialogue and constructed action in American Sign Language,” in *Sociolinguistics in Deaf Communities*, ed. Lucas C., (Washington, DC: Gallaudet University Press), 255–271.

[B86] Newport E. Supalla T. (2000). “Sign language research at the millennium,” in *The Signs of Language Revisited: An Anthology to Honor Ursula Bellugi and Edward Klima*, eds Emmorey K. Lane H., (Mahwah, NJ: Lawrence Erlbaum Associates), 103–114.

[B87] Occhino C. (2016). *A Cognitive Approach to Phonology: Evidence from Signed Languages.* Ph.D. thesis, University of New Mexico, Albuquerque.

[B88] Occhino C. (2017). An introduction to embodied cognitive phonology: claw-5 handshape distribution in ASL and libras. *Complutense J. English Stud.* 25 69–103. 10.5209/CJES.57198

[B89] Occhino C. Wilcox S. (2017). “Gesture or Sign? A categorization problem,” in *Gesture, Sign and Language: The Coming of Age of Sign Language and Gesture Studies. Behavioral and Brain Sciences*, Goldin-Meadow S. Brentari D. (Cambridge, MA: Cambridge University Press), 36–37.

[B90] Padden C. (1986). “Verbs and role-shifting in ASL,” in *Proceedings of the Fourth National Symposium on Sign Language Research and Teaching*, ed. Padden C., (Silver Spring, MD: National Association of the Deaf), 44–57.

[B91] Padden C. (1990). “The relation between space and grammar in ASL verb morphology,” in *Sign Language Research: Theoretical Issues*, ed. Lucas C., (Washington, DC: Gallaudet University Press), 118–132.

[B92] Pizzuto E. (1986). “The verb system of italian sign language (LIS),” in *Proceedings of the Second European Congress on Sign Language Research*, ed. Teervort B. T., (Amsterdam: the Institute of General Linguistics), 17–31.

[B93] Pizzuto E. (2007). “Deixis, anaphora and person reference in signed languages,” in *Verbal and Signed Languages. Comparing Structures, Constructs and Methodologies*, eds Pizzuto E. Pietrandrea P. Simone R., (Berlin: Mouton de Gruyter), 275–308.

[B94] Pizzuto E. Pietrandrea P. Simone R. (eds) (2007). *Verbal and Signed Languages. Comparing Structures, Constructs and Methodologies.* Berlin: Mouton de Gruyter.

[B95] Pizzuto E. Rossini P. Sallandre M.-A. Wilkinson E. (2008). “Deixis, anaphora and highly iconic structures: cross-linguistic evidence on American (ASL), French (LSF) and Italian (LIS) signed languages,” in *Sign Languages: Spinning and Unraveling the Past, Present and Future*, ed. Muller de Quadros R., (Petrópolis: Editora Arara Azul), 475–495.

[B96] Pizzuto E. Volterra V. (2000). “Iconicity and transparency in sign languages : a cross-linguistic cross-cultural view,” in *The Signs of Language Revisited: An Anthology in Honor of Ursula Bellugi and Edward Klima*, eds Emmorey K. Lane H., (Mahwah, NJ: Lawrence Erlbaum Associates), 261–286.

[B97] Sallandre M.-A. (2003). *Les Unités du Discours en Langue des Signes Française. Tentative de Catégorisation Dans le Cadre D’une Grammaire de L’iconicité.* Ph.D. thesis, University of Paris, Saint-Denis.

[B98] Sallandre M.-A. (2007). “Simultaneity in French sign language discourse,” in *Simultaneity in Signed Languages: Form and Function*, eds Vermeerbergen M. Leeson L. Crasborn O., (Amsterdam: John Benjamins), 103–125. 10.1075/cilt.281.05sal

[B99] Sallandre M.-A. (2014). *Compositionnalité des Unités Sémantiques en Langues Des Signes. Perspective Typologique et Développementale.* Habilitation thesis, University of Paris, Saint-Denis.

[B100] Sallandre M.-A. (2020). Comparaisons typologiques entre les langues des signes, une approche sémiologique. *Lalies* 40 9–30.

[B101] Sallandre M.-A. Balvet A. Besnard G. Garcia B. (2019). *Etude Exploratoire de la Fréquence des Catégories linguistiques dans Quatre Genres Discursifs en LSF. LIDIL. 60.* Available online at: https://journals.openedition.org/lidil/7136 (accessed September 1, 2020).

[B102] Sallandre M.-A. Di Renzo A. Gavrilescu R. (2016). “Various types of personal transfers (constructed actions) in seven sign languages,” in *Proceedings of the Theoretical Issues in Sign Language Research Conference (TISLR 12)*, (Melbourne: La Trobe University).

[B103] Schembri A. (2003). “Rethinking ‘Classifiers’ in signed languages,” in *Perspectives on Classifier Constructions in Sign Languages*, ed. Emmorey K., (Mahwah, NJ: Lawrence Erlbaum Associates), 3–34.

[B104] Sidnell J. Enfield N. (2016). “Deixis and the interactional foundations of reference,” in *The Oxford Handbook of Pragmatics*, ed. Huang Y., (Oxford: Oxford University Press), 217–239.

[B105] Sloetjes H. Wittenburg P. (2018). *ELAN (version 5.9).* Nijmegen: Max Planck Institute for Psycholinguistics.

[B106] Smith C. Lentz E. M. Mikos K. (1988). *Signing Naturally: Teacher’s Curriculum Guide. Level I.* Berkeley, CA: DawnSign Press.

[B107] Stokoe W. C. (1991). Semantic phonology. *Sign Lang. Stud.* 71 107–114. 10.1353/sls.1991.0032

[B108] Supalla T. (1978). “Morphology of verbs of motion and location in american sign language,” in *American Sign Language in a Bilingual, Bicultural Context*, eds Caccamise F. Hicks D., (Coronado, CA: National Association of the Deaf), 27–46.

[B109] Sutton-Spence R. Woll B. (1999). *The Linguistics of British Sign Language. An Introduction.* Cambridge, MA: Cambridge University Press.

[B110] Tognini-Bonelli E. (2001). *Corpus Linguistics at Work.* Amsterdam: John Benjamins.

[B111] Vermeerbergen M. (2006). Past and current trends in sign language research. *Lang. Com.* 26 168–192. 10.1016/j.langcom.2005.10.004

[B112] Wilbur R. (1979). *American Sign Language and Sign Systems: Research and Application.* Baltimore: University Park Press.

[B113] Wilcox S. (1996). Not from Jove’s brow. *Lang. Commun.* 16 179–192. 10.1016/0271-5309(96)00006-7

[B114] Wilcox S. Wilcox P. (1995). “The gestural expression of modality in American Sign Language,” in *Modality in Grammar and Discourse*, eds Bybee J. Fleischman S., (Amsterdam: John Benjamins), 135–162. 10.1075/tsl.32.07wil

[B115] Winston E. (1991). Spatial referencing and cohesion in an American Sign Language text. *Sign Lang. Stud.* 73 397–410. 10.1353/sls.1991.0003

[B116] Winston E. (1995). “Spatial mapping in comparative discourse frames,” in *Language, Gesture and Space*, eds Emmorey K. Reilly J., (Hillsdale, NJ: Lawrence Erlbaum Associates), 97–114.

[B117] Woll B. Sutton-Spence R. (2005). “Sign languages,” in *Sociolinguistics: an International Handbook of the Science of Language and Society*, eds Ammon U. Dittmar N. Mattheier K. Trudgill P., (Berlin: Walter de Gruyter), 677–683.

